# Métastase atypique du cuir chevelu et surrénalienne d'un carcinome urothelial de la vessie

**DOI:** 10.11604/pamj.2014.19.109.4874

**Published:** 2014-09-30

**Authors:** Serpos Dossou, Laurianne James, Mohammed Afif, Leila Rahali, Amine Bazine, Joelle Irigo, Etienne Ogandaga, Tayeb Kebdani, Fouad Kettani, Noureddine Benjaafar

**Affiliations:** 1 Service de Radiothérapie, Institut National d'Oncologie, CHU Ibn-sina, Université Mohammed V Souissi, Rabat, Maroc; 2 Centre d'Anatomie pathologique Nations Unies, Rabat, Maroc

**Keywords:** Métastase atypique, surrénalienne, carcinome urothelial, atypical metastasis, adrenal, urothelial carcinoma

## Abstract

Les métastases du cuir chevelu et surrénaliennes d'un carcinome urothélial de la vessie sont rares, peu de cas ont été rapportés dans la littérature, et la chimiothérapie est le traitement de choix. Nous rapportons le cas d'une femme de 60 ans qui présente un carcinome urothélial stade IV avec métastases surrénaliennes, pulmonaire et osseuses (cotes et scapula). Deux lignes de chimiothérapies ont été instaurées et la patiente bénéficia d'une radiothérapie palliative antalgique sur la scapula. Après la première ligne de chimiothérapie, une masse du cuir chevelu augmentant progressivement de volume apparue, une biopsie fut faite et le diagnostic de métastase d'un carcinome urothélial de la vessie fut confirmé par l'histologie.

## Introduction

Le cancer de la vessie est actuellement au deuxième rang des cancers urologiques aux USA et la deuxième cause de décès par cancer urogénital, le type histologique le plus fréquent est le carcinome urothélial; dans le registre des cancers de rabat 2006-2008 son incidence brute est de 4,8 pour 100000 habitants. Un tiers des tumeurs infiltrantes de la vessie vont développer des métastases, dont les sites préférentielles sont ganglionnaires, pulmonaires, hépatiques et osseuses, par contre les localisations secondaires cutanées, cérébrales, rénales et surrénaliennes sont très rares. [1] La chimiothérapie gemcitabine-cisplatine reste le traitement de première ligne de ces métastases secondaires. Nous rapportons à travers cette observation un cas de localisations secondaires atypique du cuir chevelu et surrénalienne d'un carcinome urothélial de la vessie.

## Patient et observation

**Figure 1:**
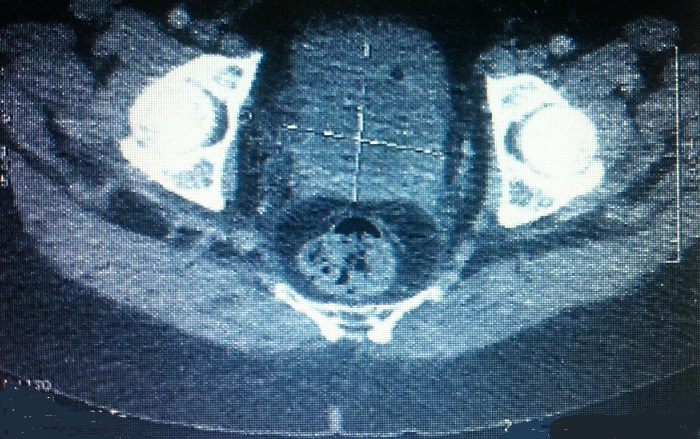
Coupe scannographique axiale montrant le processus lésionnel inter-vésico-utérin

**Figure 2:**
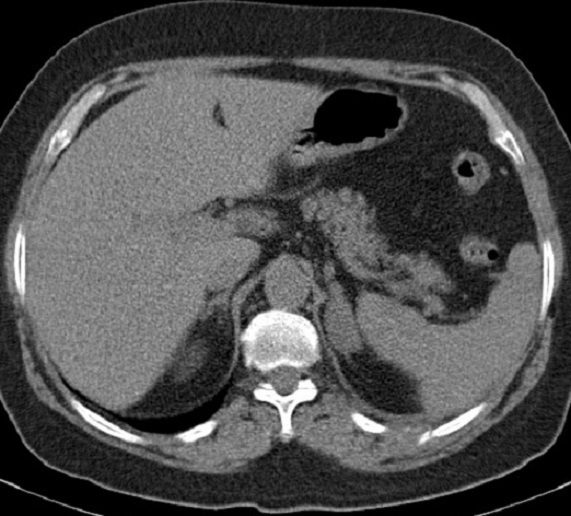
Coupe scannographique axiale montrant la métastase surrénalienne et costale

**Figure 3:**
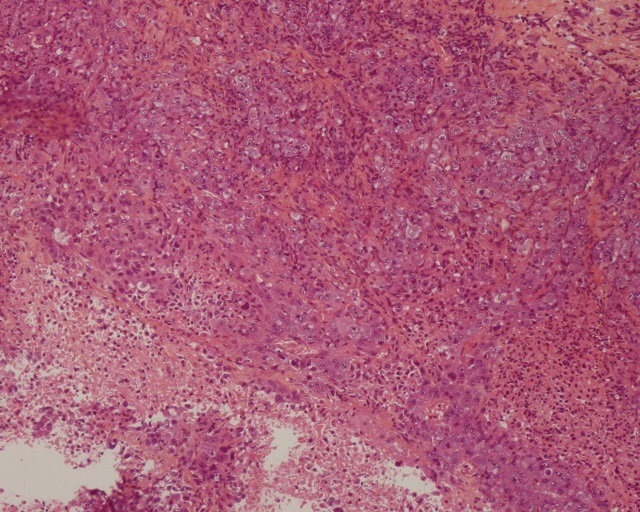
Coupe histologique montrant carcinome urothélial papillaire de grade 3, infiltrant massivement le chorion

**Figure 4:**
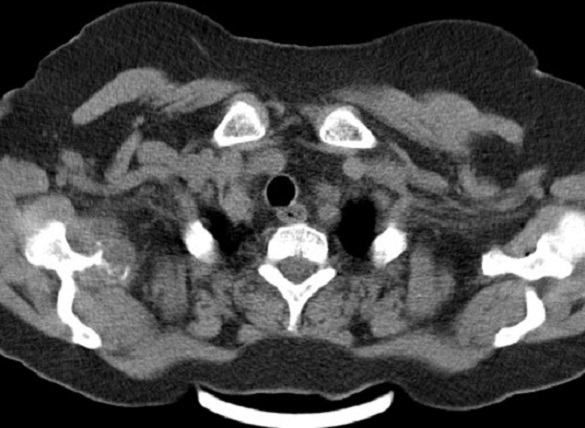
Coupe scannographique axiale montrant une lésion scapulaire secondaire

Il s'agit d'une femme de 60 ans ménopausée suivie pour cystite à répétition, avec des épisodes d'hématuries minimes, dont le bilan d'exploration révéla à l’échographie pelvienne une masse vésicale de 8 cm et à la tomodensitométrie pelvienne une lésion (Figure 1) inter-vésico-utérine associée à deux masses surrénaliennes (Figure 2) et des adénopathies lombo-aortiques. Une cystoscopie plus biopsie faite objectiva un carcinome urothélial papillaire de grade 3, infiltrant massivement le chorion (Figure 3). Le bilan d'extension fait d'un scanner thoraco-abdomino-pelvien objectiva des localisations secondaires pulmonaires osseuses (costales, et scapulaires) (Figure 2, Figure 4) et surrénalienne la classifiant ainsi stade IV.

**Figure 5:**
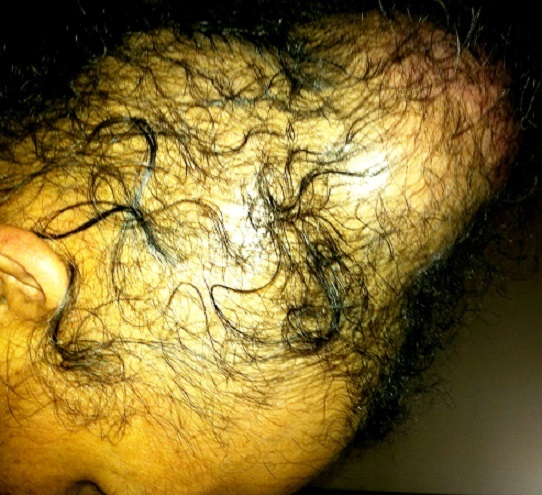
Lésion du cuir chevelu

**Figure 6:**
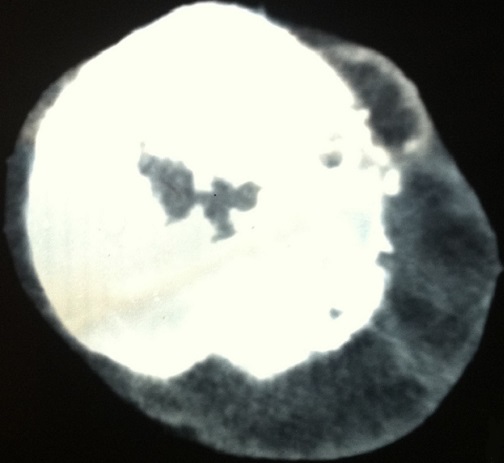
Coupe scannographique axiale montrant la lésion du cuir chevelu

**Figure 7:**
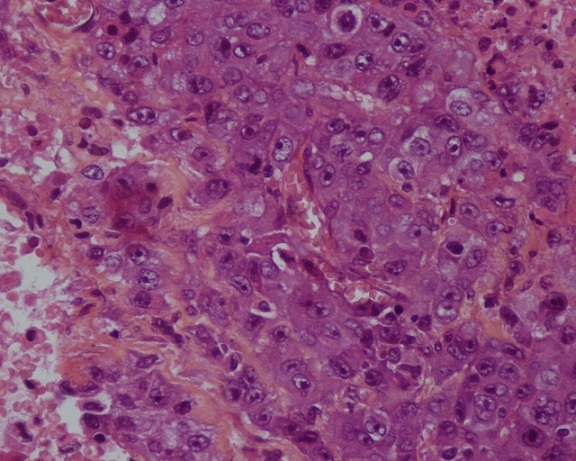
Coupe histologique montrant une prolifération tumorale d'aspect métastatique d'un carcinome urothélial peu différencié

La patiente bénéficia d'une chimiothérapie palliative première ligne type Gemcitabine en monothérapie: quartes cures à la dose de 1000mg /m^2^ (J1-J8) tous les vingt et un jours, avec bonne tolérance clinique et respect des intervalles inter-cures. L’évolution sous ce traitement a été marquée par une progression clinique et radiologique caractérisée par une accentuation des lésions surrénaliennes et scapulaires qui sont devenues douloureuses nécessitant une radiothérapie palliative antalgique sur la scapula par deux champs antérieurs et postérieur à la dose de 30 Gray avec un fractionnement de 3Gray par séance. Cette évolution a également été marqué par l'apparition d'une masse molle du cuir chevelu douloureuse augmentant progressivement de volume (15 x13 cm) (Figure 5) dont l'aspect radiologique (Figure 6) et histologique (Figure 7) est compatible avec une métastase d'un carcinome urothélial. La patiente bénéficia alors d'une chimiothérapie deuxième ligne type Paclitaxel-carboplatine et décéda un an après le début de la symptomatologie.

## Discussion

La tumeur de la vessie est diagnostiquée chez l'adulte avec un âge médian de 69 ans chez les hommes et 71 ans chez la femme, [2, 3] le carcinome urothélial est le type histologique le plus fréquemment retrouvé dans 90% des cas. Le potentiel métastatique est variable et tous les organes peuvent être envahis: le poumon, le foie et les os sont les sites fréquemment atteints [4]. Les métastases surrénaliennes sont présentes dans 14% des cas de vessie métastatique [5] et sont rarement isolées.

Les localisations secondaires cutanées d'un carcinome urothélial de la vessie sont assez rares, **Block et al** rapportent un taux d'incidence de 0,84 à 3,6% [6] et une large étude rétrospective taïwanaise ne retrouve que 2 cas de localisations secondaires cutanées sur 911 cas de tumeurs vésicales et urétérales [7]. Pour **Spector et al** les métastases cutanées sont le résultat de l'augmentation de la longévité des patients traités avec succès [8]. Quartes mécanismes de disséminations à la peau peuvent être impliqués à savoir: l'invasion directe d'une tumeur sous-jacente, l'implantation iatrogène d'une opération, l'envahissement ganglionnaire et la dissémination hématogène, [9, 10] dans notre cas la voie hématogène est la plus probable.

La présentation clinique des lésions dermatologiques métastatiques est aspécifique, elle se manifeste souvent par des nodules indolores croissants rapidement, [11] le tableau clinique le plus fréquent est une plaque ou un nodule infiltré cependant, des gonflements ulcérés, des nodules sous-cutanés, et des papules violacées ont été rapportés [10]. Les métastases viscérales et une performance statut (PS) inférieur ou égale à 70% sont reconnus comme facteurs indépendants de mauvais pronostic et conditionnent la prise en charge.

La cisplatine en monothérapie ou en association: MVAC (methotrexate, vinblastine, doxorubicine, cisplatine) reste le traitement de référence en première ligne métastatique des tumeurs vésicales, cependant à cause de leurs toxicités respectives, d'autres protocoles tels que la Gemcitabine en monothérapie ou associée à la cisplatine sont préférées. Au-delà de la chimiothérapie de première ligne métastatique, le pronostic devient de plus en plus sombre. Il n'y a pas de consensus sur la prise en charge des maladies réfractaires au cisplatine; le paclitaxel, le docétaxel, et l'ifosfamide donnent des taux de réponses entre 7 et 20%.

## Conclusion

Les localisations secondaires surrénaliennes d'un carcinome urothélial de la vessie sont rares, celles cutanées sont exceptionnelles surtout dans le cas du cuir chevelu, leurs pronostic est mauvais malgré la prise en charge.
